# Burden, anxiety and depression in caregivers of Alzheimer patients in the Dominican Republic

**DOI:** 10.1590/S1980-57642014DN84000013

**Published:** 2014

**Authors:** Martin Medrano, Rebeca López Rosario, Anyolina Núñez Payano, Natacha Reynoso Capellán

**Affiliations:** 1 MD, Professor of Geriatrics at Pontificia Universidad Católica Madre y Maestra. Director of the Unit for Clinical Research, Memory and Alzheimer (UCIMA). Chief of Geriatrics at University Hospital Jose Ma. Cabral & Baez, Santiago, Dominican Republic; 2 MD, School of Medicine, Pontificia Universidad Católica Madre y Maestra, Santiago, Dominican Republic

**Keywords:** dementia, caregiver burden, anxiety, depression

## Abstract

Alzheimer's disease (AD) has a major impact by limiting the ability to live independently. This condition of dependency involves all members of the family, particularly those who take direct care of patients. The changes that take place in caregivers' lives may alter their health and have an effect on the care of the sick.

**Objective:**

To determine the presence of burden, anxiety and depression in caregivers of Alzheimer's patients.

**Method:**

A descriptive cross-sectional study was performed in 67 family caregivers from the Alzheimer's Clinic Research Unit, Memory and Alzheimer, in the city of Santiago, Dominican Republic. Caregivers were evaluated for burden intensity with the Zarit scale and for both depression and anxiety using the respective Hamilton scales. Descriptive statistical analysis and Pearson correlation were used.

**Results:**

84% of caregivers were female, and 52% were older than 50 years. A total of 36% exhibited caregiver burden; 19% anxiety symptoms; and 43% depressive symptoms. No statistical significance was found between age, sex and number of hours of care. A significant association was found in the Pearson correlation coefficient between caregiver burden, anxiety and depression.

**Conclusion:**

Caregiver burden was associated with anxiety and depression. It is important for health professionals to include caregiver assessments in the treatment protocols of dementia. Policy should include support programs for carers.

## Abstract (pt)

A doença de Alzheimer (DA) tem um grande impacto, limitando a capacidade de viver de forma independente. Esta condição de dependência envolve todos os membros da família, particularmente aqueles que realizam o cuidado direto. As mudanças que ocorrem na vida dos cuidadores podem alterar a sua saúde e ter um efeito sobre o cuidado dos doentes.

**Objetivo:**

Determinar a presença de sobrecarga, ansiedade e depressão em cuidadores de pacientes com doença de Alzheimer.

**Método:**

Um estudo descritivo transversal foi realizado em 67 cuidadores familiares na Unidade de Pesquisa Clínica em Memória e Alzheimer, na cidade de Santiago, República Dominicana. Eles foram avaliados com relação a sobrecarga com a escala Zarit; quanto a depressão e ansiedade, com as respectivas escalas de Hamilton. Foram utilizadas análise estatística descritiva e correlação de Pearson.

**Resultados:**

84% dos cuidadores eram do sexo feminino, e 52% tinham mais de 50 anos; 36% apresentavam sobrecarga do cuidador; 19% sintomas de ansiedade; e 43% sintomas depressivos. Não foram encontradas significâncias estatísticas entre idade, sexo e número de horas de cuidados. Foi encontrada associação significativa no coeficiente de correlação de Pearson entre sobrecarga do cuidador, ansiedade e depressão.

**Conclusão:**

A sobrecarga do cuidador está associada a ansiedade e depressão. É importante que os profissionais de saúde incluam avaliações de cuidadores nos protocolos de tratamento de demência. A política deve incluir programas de apoio a cuidadores.

## INTRODUCTION

Alzheimer's disease (AD) is a degenerative, progressive and irreversible chronic brain disease. It has an insidious onset; is characterized by gradual loss of cognitive and behavioral functions, and by affection disturbances, compromising the physical, mental and social integrity of the elderly.^1^ Among the other dementias, it is the leading cause of disability in aged people, and has a major impact by reducing the capacity to live independently, which requires increasingly complex care.^2^ Thus, the importance of the family is evident in the process of providing care to the elderly, because with disease progression the demands for care and constant supervision increase, in most cases provided by a family member.^3^ Such dependence of the patient may engage all members of the family, particularly those who provide direct care. In this sense, there are two types of caregivers: the primary caregiver, who has full or most of the responsibilities for the care of the elderly at home; and the secondary caregiver, a family member, volunteer or occupational caregiver, who provides complementary assistance in activities.^4^ The changes that take place in the life of caregivers, such as lack of time, reduction of intimacy, deterioration in social life, a sense of loss of control over their own lives, may cause physical and emotional burden (anxiety, stress, and depression), acute and chronic diseases, as well as financial deterioration, affecting all activities.^5^ The physical and emotional condition of the caregiver directly affects the quality of care provided to the Alzheimer's patient. Caregiver burden may give way to patient abuse, both physical and psychological, and even neglect of the patient.^6^ Although the care of the caregiver is always considered very important by keeping a balance of attention for both patient and caregiver,^7^ evaluation of burden and possible emotional problems of caregivers is not routinely carried out by health professionals.

The Dominican Republic is a developing country with 10 million inhabitants, a GDP per capita of US$ 5,282 and a poverty rate of 33.2%.^8^ To date, no research examining the frequency of caregiver burden and the presence of anxiety and depression in this population has been published. Whether our statistics are similar to those of the region and developed countries remains unknown.

The overall objective of this study was to determine the presence of burden, anxiety and depression in caregivers of Alzheimer's patients of the Clinical Research Unit Memory and Alzheimer (UCIMA) at the Regional University Hospital José María Cabral y Baez Santiago, Dominican Republic.

## METHODS

A descriptive cross-sectional study with a primary source was conducted.

**Population.** A database with a population of 1,500 patients with Alzheimer's disease was analyzed. The diagnosis of Alzheimer's was based on the criteria of the National Institute of Neurological and Communicative Disorders and Stroke, and the Alzheimer's Disease and Related Disorders Association (NINCDS-ADRDA).^9^ The primary caregiver, having met the criteria for inclusion and exclusion, was contacted.

### Inclusion

- Of legal age, and authorized to sign the informed consent form.
- Residing at the patient's home.
- Providing more than 6 months of care giving.
- Living in the urban area of Santiago.

**Exclusion**

- Pregnancy
- Individuals with a history of depression, or taking antidepressant or psychotropic drugs
- Individuals in recent mourning (less than six months)

**Sample**. A non-probabilistic intentional sampling was conducted. Of the 1,500 patients, 525 met the criteria for inclusion-exclusion. Based on this number, the sample calculation was performed using the Epi-statcalc version 3.5.3Epi Info program. This calculation had a confidence index of 95%, and error of 5%, yielding a sample of 67 caregivers.

**Assessment procedure and instruments.** Information on burden intensity was obtained using the Zarit scale,^10^ previously validated in Spanish.^11^ This scale consists of 22 items rated on an ordinal Likert scale, ranging from "never" (value 1) to "almost always" (value 5). The items include aspects of emotional impact, social and family support, and strategies of problem management. According to the authors of the Spanish validation, a cut-off score of 46/47 differentiates 'overburden' from 'no burden', whereas the cut-off of 55/56 discriminates between "light burden" and "greater burden". The Zarit scale showed good values of convergent validity, test-retest reliability, and internal consistency in the Spanish validation.^11^

The profile and severity of depressive symptoms was assessed using the Hamilton Rating Scale for depression (17-item). This is a Likert scale with operational criteria score (0-4). Validation of the Spanish version^12^ has proven the scale's reliability, sensitivity and discriminating validity in an outpatient population.^13^ The breakpoints used were:

- Normal State: 0-7.
- Mild/ minor depression: 8-12.
- Moderate depression: 13-17.
- Severe depression: 18-29.
- Very severe depression: >30.

The Hamilton Anxiety Rating Scale was used to assess the presence and degree of anxiety. The validated Spanish version showed good internal consistency, good test-retest values, and good concurrent validity with other scales, adequately distinguishing between anxiety patients and healthy controls.^14^ The breakpoints used were:

- 0-5 no anxiety.
- 6-14 mild anxiety.
- Over 15 moderate/severe anxiety.

A pilot test with caregivers of Alzheimer's patients who met the inclusion and exclusion criteria was performed, but these were not included in the selected sample.

Data collection was performed directly by visiting the families of Alzheimer's patients. The caregiver was informed in advance by telephone. One of the researchers explained the informed consent and once the researcher was convinced the research subject was understood, the caregiver proceeded to sign. The information collection instrument, including assessment tests and explanation of informed consent took an average of 45 minutes.

**Bioethical issues.** The research project was submitted to the bioethics committee of the Faculty of Health Sciences of the Pontificia Universidad Católica Madre y Maestra (COBE-FACS), and approved with the code COBE-FACS-MED-026-3-2012-2013.

**Statistical analysis.** After completion of data collection the information was processed. To tabulate the results, a database was created in Excel 2010. The information was then exported to the program Statistical Package for the Social Sciences SPSS, version 17.0, used for recoding variables, where a plan of analysis was later performed.

The information obtained by the data collection instruments underwent statistical treatment to assess data using frequencies and percentages for qualitative variables, and the application of the Chi-square statistical test with a level of confidence of 95%, equivalent to P 0.05. Determination of the degree of association between caregiver burden, anxiety and depression, was conducted using the Pearson correlation coefficient.

## RESULTS

**Characteristics of study sample.** All caregivers were considered primary caregivers, because they were responsible for taking care of patients with AD. A total of 56 (84%) were female and 11 (16%) male. The average age was 61 years, with the age group over 50 years (52%) prevailing. Some 60% were married, 34% single, 4% divorced, and 2% widowed. The majority had elementary education (55%) and professionals represented 28%. In regards to relationship, 55% were daughters/sons, 15% spouses, 12% grandchildren, 9% brothers, and 9% had other family ties.

**Caregiver burden.** Twenty-four caregivers (36%) showed caregiver burden on the Zarit scale. Of these, 91% were female and 4 (17%) showed intense burden. Most (n 18) 75% spent between 13 and 16 hours a day caring for the sick patient. Most of the overburdened caregivers were older than 40 years (n 22) 92%, and 53% of those affected were over 50 years of age. However, these conditions had no statistical significance (P 0.737).

**Anxiety in caregivers.** A total of 19% (n 13) of caregivers had symptoms of anxiety, according to the Hamilton scale, mostly female (85%). A total of 69% were over 50 years of age, and devoted between 13 and 16 hours to the care of the sick patient. No statistical significance was found in these variables (p 0.681).

**Symptomatological profile and depression severity.** Around 43% of caregivers showed symptoms consistent with depression. Of these,72% showed mild, 10% moderate and 17% severe depression. Most depressed caregivers were female (90%) and 65% devoted from 13 to 16 hours to the care of the patient. A total of 59% was older than 51 years. There was no statistical correlation between these variables (p 0.969).

**Relationship between caregiver burden, anxiety and depression.** Pearson coefficients showed a positive correlation between anxiety and depression, plus a positive correlation between caregiver burden and anxiety, as well as between burden and depression (Table 1).

**Table 1:** Pearson correlation coefficient between caregiver burden, anxiety and depression.

Correlation		Anxiety	Depression	Burden
Anxiety	Correlation	1	0.439**	0.375**
	Significance (p)		0.000	0.002
	N	67	67	67
Depression	Correlation	0.439**	1	0.347**
	Significance (p)	0.000		0.004
	N	67	67	67
Caregiver burden	Correlation	0.375**	0.347**	1
	Significance (p)	0.002	0.004	
	N	67	67	67

** Correlation significant at 0.01 level (bilateral).

## DISCUSSION

The results of this study refer to caregivers caring for patients at home, who did not receive compensation as members of the family; therefore, burden, anxiety and depression represent only those that may be present in members of the family of these patients.

Although a psychiatric interview was not performed to evaluate specific disorders in every case, the scores of the scales applied are closely related to psychological distress situations evaluated.

Our results show that 19% of caregivers exhibited some anxiety; Cochrane et al.^5^ reported similar results with 17.5%. Other authors such as Seira et al.^16^) found anxiety in 28% of the subjects tested.

Regarding demographic variables associated with caregiver stress,^17^ 75% were women, most between 46-59 years, who spent about 13 to 24 hours a day caring for the patient. In our study, 85% were female and 69% were older than 50 years.

The symptoms profile of depression was present in 43% of caregivers in our study; Pinto et al.^18^ reported 65%. Notably, all caregivers in the present study were family members; Some caregivers expressed that this is a work entrusted by God, and it should be done with passion. We believe that this factor may play a role in the lower levels of depression in our sample.

Caregiver burden assessed by the Zarit scale was present in 36% of our evaluated sample, very low when compared with rates reported by Perez and Llibre^19^ 97%, and Alcaraz et al.^20^ who observed that 73% of women showed overburden signs.

The time spent by the caregiver in hours per day has been associated with depressive symptoms, anxiety and caregiver burden, with hours being proportional to symptoms.^21,22^ In the present study, no statistical significance was found between these variables.

In the present study, we found higher anxiety scores, higher depressive symptoms, as well as a positive correlation between caregiver burden and anxiety as well as between caregiver burden and depression. The findings of Corazza et al.^3^ suggest that depressive symptoms and anxiety are variables that can predict caregiver burden. Similar data was found by Torti et al.^1^; the presence of depression, anxiety and stress are variables that characterize the psychological distress of the caregiver and therefore overburden. Carrasco et al.^4^ found that psychological distress was significantly associated with caregiver burden assessed by the Zarit test.

Although the assessment of the economic cost in patient care was beyond the scope of this study, questions were included on it; families spent an average of 30% of their income on care. In the Dominican Republic, there are no social support services for the care of chronic patients, including Alzheimer's, with the aggravating circumstance that the social security system, dominated by private companies, excludes those over 65 years of age. We believe that the economic factor is an important aspect of psychological distress for caregivers.

It is essential to include an assessment of caregiver aspects of psychic distress and burden in the assessment and monitoring of Alzheimer's patients in order to detect these disorders early. We know that caregiver health, both physical and mental, ultimately impacts patient care.

Those responsible for social and health policies should create support mechanisms for families who have family members with chronic diseases, such as relief care, home care, day hospitals, as well as psychological and recreational support programs where physical and social activities are offered.
